# Micro-/Nanorobots Propelled by Oscillating Magnetic Fields

**DOI:** 10.3390/mi9110540

**Published:** 2018-10-23

**Authors:** Hao Yu, Wentian Tang, Guanyu Mu, Haocheng Wang, Xiaocong Chang, Huijuan Dong, Liqun Qi, Guangyu Zhang, Tianlong Li

**Affiliations:** 1 State Key Laboratory of Robotics and System, Harbin Institute of Technology, Harbin 150001, China; yu1997hao@gmail.com (H.Y.); 1150850205@stu.hit.edu.cn (W.T.); muguanyu@gmail.com (G.M.); wanghaocheng1989@126.com (H.W.); xiaocongchang@hotmail.com (X.C.); dhj@hit.edu.cn (H.D.); qiliqun.hit@163.com (L.Q.); 2 Department of Analytical, Physical and Colloidal Chemistry, Institute of Pharmacy, Sechenov University, 119991 Moscow, Russia

**Keywords:** micro-/nanorobots, oscillating magnetic fields, propulsion mechanisms, fabrication techniques

## Abstract

Recent strides in micro- and nanomanufacturing technologies have sparked the development of micro-/nanorobots with enhanced power and functionality. Due to the advantages of on-demand motion control, long lifetime, and great biocompatibility, magnetic propelled micro-/nanorobots have exhibited considerable promise in the fields of drug delivery, biosensing, bioimaging, and environmental remediation. The magnetic fields which provide energy for propulsion can be categorized into rotating and oscillating magnetic fields. In this review, recent developments in oscillating magnetic propelled micro-/nanorobot fabrication techniques (such as electrodeposition, self-assembly, electron beam evaporation, and three-dimensional (3D) direct laser writing) are summarized. The motion mechanism of oscillating magnetic propelled micro-/nanorobots are also discussed, including wagging propulsion, surface walker propulsion, and scallop propulsion. With continuous innovation, micro-/nanorobots can become a promising candidate for future applications in the biomedical field. As a step toward designing and building such micro-/nanorobots, several types of common fabrication techniques are briefly introduced. Then, we focus on three propulsion mechanisms of micro-/nanorobots in oscillation magnetic fields: (1) wagging propulsion; (2) surface walker; and (3) scallop propulsion. Finally, a summary table is provided to compare the abilities of different micro-/nanorobots driven by oscillating magnetic fields.

## 1. Introduction

Locomotion of synthetics micro-/nanorobots is so fundamental and practical that it has inspired major research efforts over the past decade [1,2,3,4,5]. Self-propelled micro-/nanorobots are micro-/nanoscale devices that convert light energy, thermal energy, and other energies into kinetic energy and propulsion force [6,7,8,9]. Their micro size determines that they are difficult to design, manufacture, and even navigate. Only recently have nanotechnology, material science, physics, chemistry, medical science, and automatic control merged together to contribute to the development of micro-/nanorobots. Micro-/nanorobots, employed as power devices of micro-/nano mechanical systems that are capable of performing complex tasks, have great potential to revolutionize emerging topics in multidisciplinary nanotechnology [10,11], manufacturing [12,13,14,15], noninvasive surgery [16,17], targeted drug delivery [18,19,20,21,22,23,24], cell manipulation and isolation [25,26], bioimaging or biosensing [27,28,29,30], environmental monitoring and remediation [31,32,33,34,35].

Different energy sources are used for the propulsion of synthetic micro-/nanorobots [36,37,38,39,40]. The most common mechanism is chemical propulsion, which can convert local chemical energy into microbubbles or a concentration gradient to locomote micro-/nanorobots in a fluid with a low Reynolds number [41,42,43,44,45,46,47,48,49,50]. For example, hydrogen peroxide is widely used for the propulsion of bimetal nanowire swimmers [51,52,53,54], Janus micro-/nanoswimmers [55,56,57,58,59,60,61], and tubular micro-/nanorockets [62,63,64,65,66,67,68,69]. However, the incomplete fuel degradation of chemical propelled micro-/nanorobots hinders the further development of practical and routine biomedical applications, especially targeted drug delivery, cell manipulation, and isolation. Fuel-free synthetic micro-/nanomotors driven by external stimuli such as light [67,70,71,72,73,74,75,76,77,78] magnetic [79,80,81,82,83,84,85,86], ultrasonic [87,88,89], or electric fields [90,91] are widely reported for their efficiency in propulsion in a high-viscosity or high-ionic-strength biological environment. Taking advantage of the on-demand motion control, long lifetime, and great biocompatibility, magnetic propelled micro-/nanorobots have exhibited considerable promise for diverse potential biomedical and biosensing studies [7,92,93,94].

**Figure 1:**
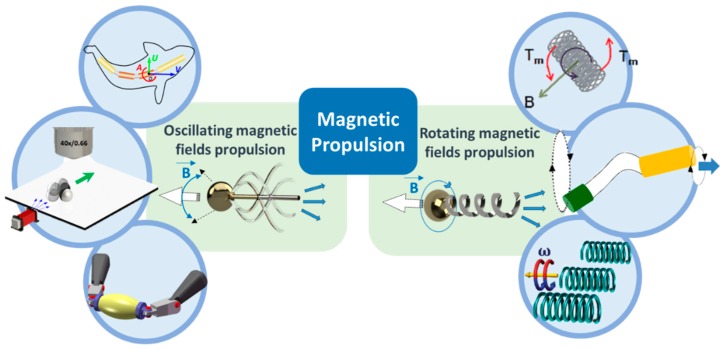
The categories of magnetic propulsion. Adapted with permission from Reference [84], copyright Small 2016; adapted with permission from Reference [85], copyright Advanced Functional Materials 2018; adapted with permission from Reference [83], copyright Nano Letter 2017; adapted with permission from Reference [100], copyright Advanced Materials 2013; adapted with permission from Reference [101], copyright Journal of the American Chemical Society 2010; adapted with permission from Reference [102], copyright Nanoscale 2014.

According to the propulsion mechanisms, magnetic propelled micro-/nanoswimmers can be further categorized into two groups (Figure 1). The first type is propelled by magnetic fields called rotating magnetic fields (direction change with time), which are inspired by bacterial flagella [95,96,97,98]. Another type is powered by oscillating magnetic fields (strength change with time) which rely on asymmetrical shape deformation to escape the constraints of Purcell’s famous “scallop theorem” [99]. These two types of micro-/nanorobots both have distinctive advantages and application objects, which are primarily affected by their structure and locomotion mode. Therefore, this review will mainly introduce related fabrication techniques and propulsion approaches to guide future designs for micro-/nanorobots. The content will specifically focus on those fuel-free micro-/nanorobots propelled by oscillating magnetic fields, as inspiring breakthroughs in this research field concerning bionics in the microscale have been made in recent years, such as the imitation of the locomotion mechanisms of fish [85].

## 2. Fabrication Techniques

### 2.1. Electrodeposition

**Figure 2:**
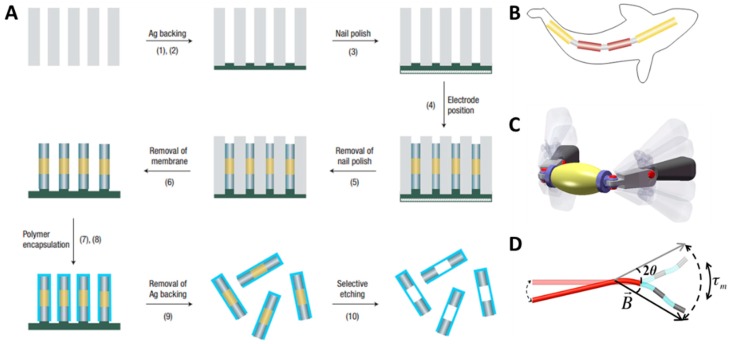
Membrane template-assisted electrodeposition of nanowires. (**A**) Preparation procedure of flexible metallic nanowires with polyelectrolyte hinges after membrane template electrodeposition. Adapted with permission from Reference [103], copyright Nature Nanotechnology 2007. (**B**) Schematic illustration of a fish-like nanoswimmer. Adapted with permission from Reference [84], copyright Small 2016. (**C**) Model of a freestyle magnetic nanoswimmer. Adapted with permission from Reference [83], copyright Nano Letter 2017. (**D**) Schematic representation of a magnetic multilink nanoswimmer. Adapted with permission from Reference [104], copyright Nano Letter 2015.

Electrodeposition can easily be realized without expensive instruments and strict experimental conditions, and enables the fabrication of a variety of structures with different materials. Therefore, this technique is a widespread approach applied to micro-/nanorobots driven by oscillating magnetic fields. In 2007, Mirkovic et al. [103] first utilized this technique to make flexible metal multilink nanorods (Figure 2A). This fabrication method allows researchers to change the length and material of each segment, so it has great versatility. After Mirkovic’s study, some other researchers gradually developed several types of multilink nanoswimmers, such as fish-like nanoswimmers (Figure 2B), freestyle magnetic nanoswimmers (Figure 2C), and magnetic multilink nanoswimmers (Figure 2D) [83,84,104]. The most important part of a multilink nanoswimmer is the hinges between the two segments, which determine the flexibility and the propulsion efficiency of the nanorobots. To manufacture this part perfectly, Mirkovic et al. manufactured the special hinges by layer-by-layer assembly, which will be introduced in the next section.

### 2.2. Self-Assembly

The self-assembly technique can help researchers combine various disordered elements to prepare an organized structure through a spontaneous reorganization process. This process can be simply divided into layer-by-layer (LbL) self-assembly and the assembly of micro-/nanoparticles.

**Figure 3:**
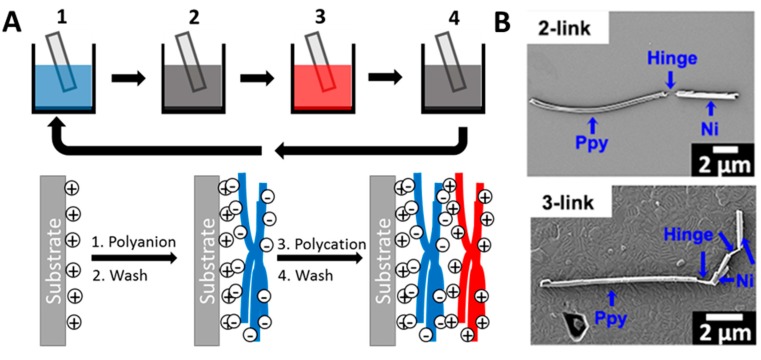
(**A**) Schematic illustration of the layer-by-layer assembly process. (**B**) SEM images of 2-link and 3-link nanoswimmers. Adapted with permission from Reference [104], copyright Nano Letter 2015.

LbL is a manufacturing technique involving the deposition of alternating layers of oppositely charged materials to fabricate a multilayer structure (Figure 3A) [105]. It has the ability to easily and cheaply incorporate distinctive materials, such as small organic molecules, inorganic compounds, macromolecules, and colloids. Researchers have used this technique to fabricate a series of rube and particle micro-/nanorobots. In order to make a soft hinge, Mirkovic et al. [103] encapsulated barcoded metal nanorods with layer-by-layer electrostatically self-assembled polyelectrolyte multilayers. Subsequently, they selectively etched hard metal segments to expose soft polymer hinges. Similarly, in 2015, Jang et al. [104] combined electrodeposition, layer-by-layer deposition, and selective etching to produce multilink nanorobots. They used polypyrrole to make a long tail, and manufactured ferromagnetic nickel rods by iron and nickel. Using this method, they tried out three different multilink nanoswimmers which were termed as 1-link, 2-link, and 3-link, respectively (Figure 3B).

Micro-/nanorobots fabricated by the assembly of micro-/nanoparticles are composed of several different particles or molecules through a series of chemical assembly processes. In 2005, Dreyfus et al. [106] fabricated a microrobot called a microscopic artificial swimmer, which was the first microrobot driven by an oscillating magnetic field. This microrobot consists of a red blood cell and super paramagnetic micro colloids (φ1 mm) linked by DNA. The flexibility depends on the length, the number of the DNA linkers, and the particle diameter. During the fabrication process, researchers synthesized biotinylated double-stranded DNA and purified the solution. At the same time, they also prepared superparamagnetic particles with streptavidin grafted onto their surface and in the red blood cells solution. After mixing these three constituents in specific proportions, they successfully fabricated these flexible magnetic filaments.

### 2.3. Electron Beam Evaporation

**Figure 4:**
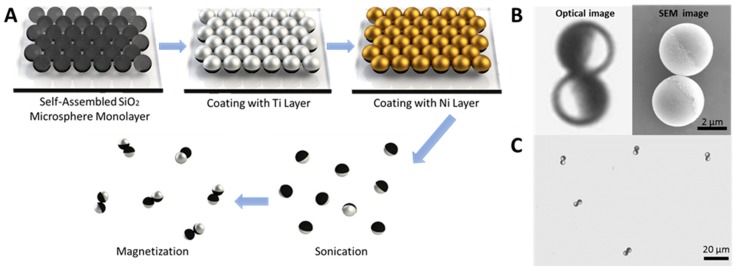
(**A**) Schematic illustration of the electron beam evaporation process. (**B**) Magnified optical microscopy image and the corresponding SEM image of a microdimer, highlighting its staggered shape and the magnetic hemispheres (dark under the optical microscope, bright under the SEM). (**C**) Optical microscopy image of a few representative microdimers after magnetization. Adapted with permission from Reference [85], copyright Advanced Functional Materials 2018.

Electron beam evaporation is a type of conventional physical vapor deposition technique which can incorporate desired materials into micro-/nanorobots to perform special functions. It is mostly used to coat nonmagnetic particles with a magnetic material layer. Li et al. [85] applied it to fabricate a Janus microdimer surface walker. This is a type of new micro-/nanorobot which includes two Janus microspheres that are magnetized so as to be propelled by oscillating magnetic fields. Using electron beam evaporation, Li et al. coated half of each microsphere with a desired nickel layer (Figure 4A,B). In 2014, Khalil et al. [107] also used this technique to develop a sperm-shaped microrobot whose head could be affected by controlled oscillating weak magnetic fields. Firstly, MagnetoSperm bodies were fabricated by developing an SU-8 layer printed MagnetoSperm. It was patterned by ultraviolet (UV) exposure in RER600 (ARCH Chemicals, Basel, Switzerland) after the pre-bake. Then, deposited by electron beam evaporation and subsequently lifted-off, a 200-nm-thick cobalt-nickel layer coated the surface of the MagnetoSperm’s head (Figure 4C).

### 2.4. Three-Dimensional Direct Laser Writing

**Figure 5:**
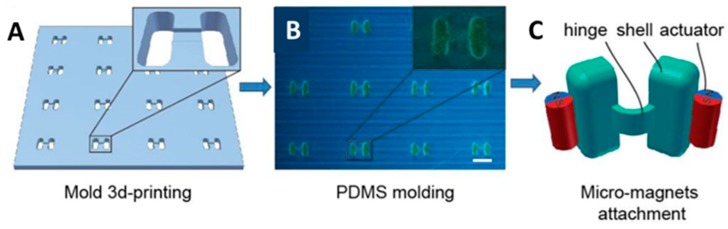
(**A**) Fabrication of a microrobot mold via three-dimensional (3D) printing. (**B**) Image of the fabrication of PDMS (polydimethylsiloxane) shells in the mold. (**C**) Schematic illustration of scallop swimmers. Adapted with permission from Reference [108], copyright Nature Communications 2014.

Three-dimensional (3D) direct laser writing (DLW) is a mature technique which is used to fabricate complex structures. Therefore, it is applied by some researchers to manufacture micro-/nanorobots or their molds. The study of Qiu et al. [108] introduced a microrobot which is similar to a scallop, which was readily constructed by 3D printing and the micro-molding technique. First, researchers printed the negative molds of the micro-scallop using a 3D printer (Figure 5A). After that, these molds were filled with a PDMS (polydimethylsiloxane) solution to make the PDMS shells (Figure 5B). The hinge between two shells was fabricated at the same time, which is narrower and thinner then shells. This design is able to decline the elastic force for the microrobot. Finally, released from the molds, the shells were attached to two neodymium micro-magnets (Figure 5C). Using this technique, researchers can fabricate a number of microrobots efficiently.

## 3. Propulsion Mechanisms

### 3.1. Wagging Propulsion

**Figure 6:**
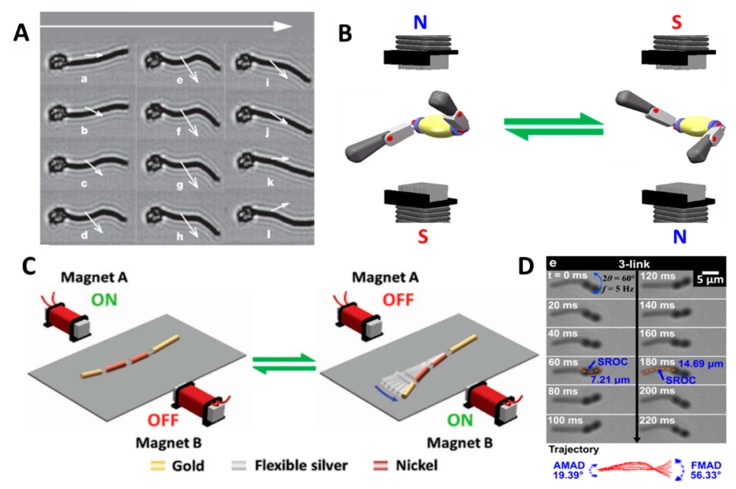
(**A**) Beating pattern of the motion of a magnetic flexible filament attached to a red blood cell. Adapted with permission from Reference [106] copyright Nature 2005. (**B**) Schematic showing the magnetic setup for propulsion along with the vibrating magnetic field. Adapted with permission from Reference [83], copyright Nano Letter 2015. (**C**) Magnetic propulsion of an artificial nanofish using a planar oscillating magnetic field. Adapted with permission from Reference [84], copyright Small 2016. (**D**) Image sequences of 3-link swimmers’ wagging motion. Adapted with permission from Reference [104], copyright Nano Letter 2015.

Inspired by the swimming mechanisms of fish or bacterial flagellum, researchers proposed wagging propulsion as an efficient propulsion mode for micro-/nanomotors. Moreover, in an oscillating magnetic field, the magnetic part of a micro-/nanometer tends to change its status and follow the change of the field strength. Therefore, wagging propulsion has been widely applied to drive micro-/nanomotors in past studies. Dreyfus et al. [106] used a magnetic 24-μm-long filament to transport red blood cells and record their movement with a fast camera. In order to realize wagging propulsion, they set up a propulsion magnetic field which included a homogeneous static field **B**_x_ = *B_x_***x** and a sinusoidal field **B**_y_ = *B_y_*sin(2πft)**y**. With the transverse field **B**_y_ oscillating, the tail of the filament would swing and propel the micro-/nanorobots along **B**_x_ (Figure 6A). Similarly, in 2014 the sperm-shaped microrobot designed by Khalil et al. was also propelled by two types of magnetic fields [107]. One type generated uniform magnetic fields in any direction. The other set up oscillating magnetic fields to cause the head to vibrate. The oscillating magnetic field pushed the MagnetoSperm to wag his long tail like a sperm. By open loop control and closed loop control, researchers found that the MagnetoSperm moved faster in water when the oscillating magnetic field frequency became higher. However, the result reversed when they propelled the MagnetoSperm on the water surface; it slowed down with the increasing magnetic field frequency.

What is more, Jang et al. [104] first fabricated wire multilink nanorobots propelled by two sets of coplanar, opposing coil pairs situated at a 90-degree offset. These two pairs of coils imposed a planar oscillating magnetic field on the nanorobots by generating two sinusoidal oscillating fields. Comparing three types of nanoswimmers with different numbers of links, they observed that these nanoswimmers have different swimming and wagging statuses. It was found that the 3-link swimmers could move faster and more efficiently than the 1- and 2-link swimmers, because an S-like motion was achieved by the tail of the 3-link swimmers (Figure 6D). This feature is achieved thanks to the greater number of links, indicating a higher degree of freedom. Therefore, they argued that the increased freedom can increase the speed of this nanoswimmer, when propelled at the optimum frequency.

In 2016, Li et al. [84] developed a new fish-like body-deformable multilink artificial nanoswimmer composed of one gold head, two nickel bodies, and one gold caudal fin. These four segments were connected by three flexible porous silver hinges. The nickel elements are triggered by the oscillating magnetic fields and carry out a fan-shaped swing, which drives the gold segments to wag, resulting in a movement that resembles the body and caudal fin (BCF) mode propulsion of fish (Figure 6C). As a result, this nanoswimmer is able to move faster than most of the other artificial magnetic propellers (up to ≈0.6 body length per cycle).

Luffing propulsion is a special wagging propulsion, which commonly exhibits an efficient nonplanar freestyle stroke. Li et al. [83] realized this locomotion and manufactured an interesting and creative freestyle magnetic nanoswimmer consisting of a central gold body and two side nickel arms. Due to the flexible porous silver hinges between two segments, the freestyle magnetic nanoswimmer is able to move in luffing mode, like a freestyle swimmer, which is much faster than the fish-like nanoswimmer. Virtually, the oscillating magnetic field exerts a torque on the magnetic Ni arms and wobbles both arms, which enables the whole swimmer’s body to move forward by luffing propulsion (Figure 6B).

### 3.2. Surface Walker

**Figure 7:**
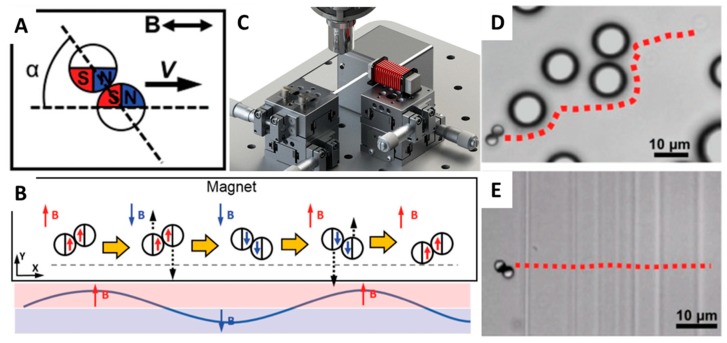
(**A**) When exposed to a magnetic field, the dimer immediately aligns its major axis with the field direction at an angle α of 47.6 ± 2.6°, determined by the dimer geometry. (**B**) Schematic showing the propulsion mechanism of microdimer surface walkers under an oscillating magnetic field. (**C**) Schematic of the experimental setup in which a magnetic dimer moves along the magnetic field direction, away from the magnet. (**D**) A microdimer surface walker bypasses several polystyrene microspheres. (**E**) A microdimer surface walker crosses continuous cracks on a glass surface. Adapted with permission from Reference [83], copyright Advanced Functional Materials 2018.

In 2008, Tierno et al. [109] reported a new propulsion mechanism: the combination of special colloidal particles linked by DNA was observed to be able to rotate while clinging to a float flat, like a surface walker. This propulsion mode allowed micro-/nanomotors to swim efficiently and even overcome obstacles. Following this, researchers also fabricated many other distinctive surface walkers, including a chain of self-assembled colloidal rotors, nickel nanowires, or cylindrical-shaped microrobots [100,101,102,103,104,105,106,107,108,109,110,111], all propelled by a rotating magnetic field. In 2018, Li et al. [85] invented a micro-/nanorobot called a Janus microdimer surface walker. First, they fabricated some silica microspheres (3 µm in diameter) and coated half of their surface with a 15-nm-thick layer of nickel to obtain Janus microspheres. After that, these separate Janus microspheres were forced to connect with each other by magnetic dipole-dipole interactions (Figure 7A,C). Interestingly, the two Janus spheres were found to be able to periodically roll under an external oscillating magnetic field and did not roll independently but rather in a coordinated and hinged fashion (Figure 7B). Furthermore, this micromachine was proved to be able to round small objects as well as overcome cracks obstructing their paths (Figure 7D,E).

### 3.3. Scallop Propulsion

**Figure 8:**
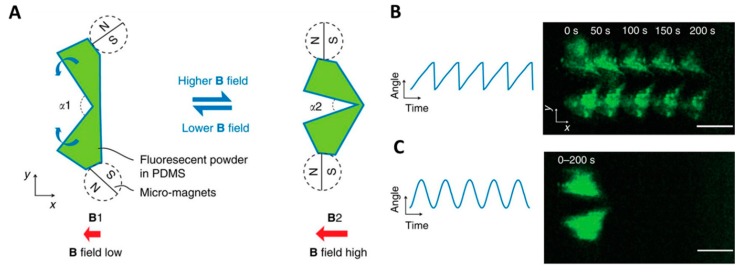
(**A**) Schematic drawing of a micro-scallop from the top view. The green shapes illustrate the opening and closing shape change of the micro-scallop when actuated by an external magnetic field. (**B**) Forward net displacement of the micro-scallop in a shear thickening fluid and asymmetric actuation (blue curve). (**C**) Forward net displacement of the micro-scallop in a shear thickening fluid with symmetric actuation (blue curve). Adapted with permission from Reference [108], copyright Nature Communications 2014.

The results of past research have proved that movement in a low-Re fluid generally requires the nonreciprocal actuation of the nanoswimmers. If this is not achieved, the net displacement of the periodic motion will be zero. Qiu et al. discovered a new method to meet this requirement. In order to break reciprocity and propel scallop swimmers efficiently in viscous fluids, these researchers applied asymmetric oscillating magnetic fields whose strength determined the opening angle α of the micro-scallop (Figure 8A) [108]. They discovered that, in either a thickening fluid or a thinning fluid, the forward net displacement of the scallop swimmers would be zero if the oscillating magnetic fields were symmetrical, such as a sine wave (Figure 8B,C). Furthermore, asymmetric actuation in a Newtonian fluid would also result in a net displacement of zero. Finally, after analysis, researchers concluded that there were three necessary conditions to propel nanoswimmers: a clear fore-aft asymmetry, time-asymmetric actuation, and coupling to a non-Newtonian fluid rheology.

In order to compare the propulsion performance of the above micro-/nanorobots driven by oscillating magnetic fields, as shown in Table 1. In the table, we show the maximum dimensional speeds $U_{\max}$ and the maximum dimensionless speed ${\bar{U}}_{\max}=U/Lf$ (*L*—the corresponding characteristic, *f*—the actuation frequency). Micro-/nanorobots move at different speeds when the magnetic field frequency changes, and there is an optimum frequency at which the speed can reach the top value. Generally, multilink nanoswimmers have a more efficient locomotion, which can be further improved by a greater number of segments and longer arms.

We have reviewed the propulsion mechanisms and fabrication methods of micro-/nanorobots propelled by oscillating magnetic fields. Micro-/nanorobots driven by oscillating magnetic fields, like most magnetically propelled micro-/nanorobots, not only have good biocompatibility, but also exhibit some unique characteristics. The recent research on their production has enriched the category of magnetically propelled micro-/nanorobots and increased the possibility of their wider application. These different propulsion approaches suit different micro-/nanorobot requirements, so researchers commonly choose them according to the micro-/nanorobots’ specific properties.

## 4. Conclusions

In conclusion, the determining factor in the improvement of micro-/nanorobots’ performance is not the type of magnetic field, but rather the micro-/nanorobot design, concerning the structure and locomotion mode. For instance, multilink nanoswimmers have shown many possibilities for innovation in design, and several breakthroughs have been achieved for their fabrication. However, it is obvious that their relevant applications are finite, and remain at a state of technology accumulation. The principal future direction in the biomedical micro-/nanorobots field is to design biocompatible micro-/nanorobots that can be fabricated efficiently and at a large scale, as well as functionalized by easy and low-cost approaches. As the technical bottleneck is constantly broken through, magnetic propelled micro-/nanomotors are expected to have profound and general applications on a variety of fields, such as in the treatment of cancer, thrombosis, and oculopathy.

## Figures and Tables

**Table 1:** Propulsion performance of micro-/nanorobots propelled by oscillating magnetic fields.

Type of Micro-/Nanorobots	Schematic or Image	Body Length (μm)	Maximum Dimensional Speed *U*_max_ μ(m/s)	Maximum Dimensionless Speed *U*_max_ (Body Length/s)	Maximum Dimensionless Speed ${\bar{U}}_{\max}$
Microscopic artificial swimmers [106]	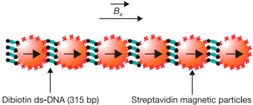	-	-	-	0.09
Scallop swimmers [108]	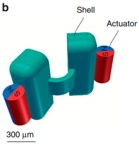	300	5.2	0.017	-
Magnetic multilink nanoswimmers [104]	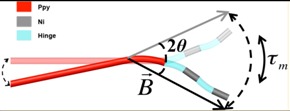	15.5	14.4	0.9	0.09
Fish-like nanoswimmers [84]	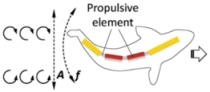	4.8	30.9	6.9	0.63
Freestyle magnetic nanoswimmers [83]	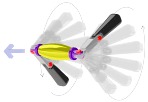	4.8	59.6	12	-
Janus microdimer surface walkers [85]	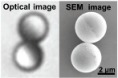	10	20	2	-
